# Intensity‐modulated radiotherapy and volumetric‐modulated arc therapy for malignant pleural mesothelioma after extrapleural pleuropneumonectomy

**DOI:** 10.1120/jacmp.v14i4.4130

**Published:** 2013-07-08

**Authors:** Jerome Krayenbuehl, Oliver Riesterer, Shaun Graydon, Peter Dimmerling, Stephan Kloeck, Ilja F. Ciernik

**Affiliations:** ^1^ Department of Radiation Oncology University Hospital Zurich Zurich Switzerland; ^2^ Department of Radiation Oncology City Hospital Dessau Germany; ^3^ Center for Clinical Research Zurich University Hospital Zurich Switzerland

**Keywords:** mesothelioma, extrapleural pleuroneumectomy, intensity‐modulated radiotherapy, volumetric‐modulated arc therapy, air cavity

## Abstract

Radiotherapy reduces the local relapse rate after pleuropneumonectomy of malignant pleural mesothelioma (MPM). The optimal treatment technique with photons remains undefined. Comparative planning for intensity‐modulated radiotherapy (IMRT) and volumetric‐modulated arc therapy (VMAT) was performed. Six MPM patients with significant postoperative intrathoracic air cavities were planned with IMRT and VMAT. A dose comparison for the targets and organ at risks (OAR) was performed. Robustness was assessed in respect to the variation of target dose with change in volume of air cavities. VMAT reduced the dose to the contralateral lung by reducing the volume covered by 13 Gy and 20 Gy by a factor 1.8 and 2.8, in respect to IMRT (p=0.02). Dose distribution with VMAT was the most stable technique in regard to postsurgical air cavity variation. For IMRT, $V_{90},V_{95}$, and the minimal target dose decreased by 40%, 64%, and 12% compared to 29%, 47%, and 7% with VMAT when air cavity decreased. Two arcs compared to one arc decreased the dose to all the organs at risk (OAR) while leaving PTV dose coverage unchanged. Increasing the number of arcs from two to three did not reduce the dose to the OAR further, but increased the beam‐on time by 50%. Using partial arcs decreased the beam‐on time by 43%. VMAT allows a lower lung dose and is less affected by the air cavity variation than IMRT. The best VMAT plans were obtained with two partial arcs. VMAT seems currently the most suitable technique for the treatment of MPM patients when air cavities are remaining and no adaptive radiotherapy is performed.

PACS number: 87.55.D‐

## I. INTRODUCTION

Malignant pleural mesothelioma (MPM) is an aggressive tumor with a high mortality rate and a survival rate of 38% after two years and 15% after five years.^(1)^ The role of RT after extrapleural pneumonectomy (EPP) is to reduce local failure.^2^, ^3^ The dose to the hemithorax is typically limited to 45 Gy with a boost to 55 Gy^2^, ^4^ due to the adjacent dose limiting structures, such as the lung, kidney, spinal cord, liver, and heart. However, the tumor control rates after EPP, chemotherapy, and radiotherapy remains poor.^(5)^ In order to enhance tumor control, dose escalation is an option if dose conformity is improved (e.g., proton therapy has been proposed for dose escalation due to the low dose delivered to the contralateral lung, heart, and kidney).^(6)^

However, access to proton therapy (PT) remains limited and proton techniques are hampered by postsurgical air cavities. After EPP, the ipsilateral hemithorax contains variable amounts of air, which subsequently are replaced by fibrous tissue. These cavities decrease over time, reducing drastically the dose to the target for PT which would decrease tumor control and counteract any potential benefit of an eventual dose escalation.

In this study, an evaluation of a new RT technique, volumetric‐modulated arc therapy (VMAT) was performed for the treatment of MPM patients. The optimal number of arcs and degree of rotation were evaluated for the treatment of MPM patients with VMAT. Postoperative IMRT and VMAT were compared in order to define potential advantages of each technique: dosimetric benefit and treatment time. Another goal was to evaluate the dose variation of VMAT and IMRT in respect to the variation of air cavities in the resected lung, and to evaluate for which air cavity variation adaptive planning is required. This was evaluated in respect to the change of dose in the target.

## II. MATERIALS AND METHODS

Between 2004 and 2011, 16 patients diagnosed with MPM were treated with external radiotherapy at the Zurich University Hospital, Switzerland. All of these patients received preoperative chemotherapy followed by extrapleural pleuropneumonectomy (EPP) and modulated radiotherapy. Six of 16 patients had air cavities exceeding 100 cm^3^ when the treatment planning computer tomography (CT) was performed and were selected for the present study. All CTs have been performed in supine head‐first position. Five patients were male and one female. Five patients presented with right‐sided MPM and one patient with left‐sided MPM. Two of these six patients were treated with VMAT and four patients were treated with IMRT. All patients were planned with IMRT and VMAT, two patients prospectively and four patients retrospectively. The comparison between IMRT and VMAT was based on dose distribution, treatment time, and robustness of the techniques in respect to the effect of variation of the air cavity on the dose distribution.

Target volume definition was obtained as described previously in detail.^(5)^ All patients were planned and treated with 26×1.75Gy(45.5Gy) to the planning target volume two (PTV2) including a simultaneously integrated boost of 26×2.15Gy(55.9Gy) to the PTV1. Due to the difficulty of the algorithm to optimize correctly the dose in air cavity, a PTV2* was defined for evaluation purpose only as PTV2 without air cavity.

### A. IMrt and VMAt treatment

The technique used for IMRT was described previously^(6)^ and followed the recommendation from Allen et al.^(7)^

The VMAT plans were performed with two clockwise arcs of 205° ranging from 180° to 25° for right‐sided tumors and from 335° to 180° for left‐sided tumors. The isocenter was placed in the middle of the PTV2. The partial‐arc technique was used in order to avoid entrance dose to the contralateral lung. The collimator angles were set to 355° and 5°.

The patient with the PTV volume closest to the mean PTV volume of all six MPM patients was chosen to assess the optimal number of arcs and the rotation angle required to treat MPM patients. Plans with one, two, or three full arcs (360°) or partial arcs (205°) were performed in order to assess the optimal number of arcs and gantry rotation. When one arc was used, the collimator angle was set to 5°. If a second arc was added (respectively a third arc), the collimator angle was set to 355° (respectively 10°).

### B. dose calculation and delivery

Calculation and optimization were performed for IMRT and VMAT using an inverse treatment planning system (HELIOS, Eclipse V8.9 with AAA 8.9.08 algorithm, Varian Medical System, Palo Alto, CA). One patient was calculated with Acuros XB (Varian Medical System) in order to quantify the dose distribution difference between one of the most advanced algorithm (Acuros) and AAA, the algorithm used clinically.^(8)^ Treatments were delivered on a 6 MV photon linear accelerator (Trilogy, Varian Medical Systems) equipped with a Millennium multileaf collimator with 120 leaves. Patient positioning was verified with either cone‐beam CT (CBCT) or with two orthogonal kV images.

Pretreatment dose verification was performed with a cylindrical PMMA phantom having two perpendicular planes of 1069 diodes (Delta^4^; ScandiDos Inc., Uppsala, Sweden). The verification of the plan had to reach a gamma score of 95% (3% dose difference, 3 mm distance to agreement) before patient irradiation.

### C. dose volume constraints, plan, and treatment comparison

The dose constraints chosen were published previously^(6)^ and are summarized in Table 1. DVHs were calculated for the PTVs and OARs for each plan. Target dose distributions were evaluated according to the volume covered by $80\%,(V_{80}),85\%(V_{85}),90\%(V_{90}),95\%(V_{95})$ and 105% ($V_{105}$) of the prescribed dose and to the minimal dose ($D_{99}$) and maximal dose ($D_{1}$). The dose to OARs were evaluated according to mean dose, and organ‐specific tolerance levels such as the volume covered by 15 Gy ($V_{15}$) for kidneys, maximal dose to the spinal cord ($D_{1}$), and $V_{5},V_{13}$, and $V_{20}$ for contralateral lung.^1^, ^9^, ^10^

The treatment time, as well as the number of monitor units needed for one fraction, were evaluated for IMRT and VMAT.

**Table 1:** Dose objectives and mean dose‐volume histogram results from six IMRT and corresponding VMAT plans, respectively

	*Objectives*	*IMRT*	*VMAT*	*p‐test* ^a^
$\text{PTV}1(V_{95})(\%)$		94.7±2.1	94.2±1.3	0.39
$\text{PTV}1(V_{105})(\%)$		4.5±3.5	6.3±2.0	0.49
$\text{PTV}1(D_{99})(\%)$	95	90.9±2.8	91.4±1.3	0.13
$\text{PTV}1(D_{1})(\%)$	107	106.7±1.8	106.8±0.7	0.18
$\text{PTV}2*(V_{95})(\%)$	95	94.8±3.0	95.3±2.4	0.38
$\text{PTV}2*(V_{105})(\%)$	10	20.1±9.0	18.3±8.8	0.21
Lung mean dose (Gy)	8.5	5.2±0.9	4.6±1.5	0.23
Lung V (%)	50	32.4±18.3	40.8±13.6	0.23
Lung V (%)	20	6.1±1.8	3.3±1.4	0.02
Lung V (%)	10	2.5±1.2	0.9±1.1	0.02
Contralateral kidney mean dose (Gy)	12	3.6±1.2	3.8±1.8	0.47
Contralateral kidney V (%)	20	0	0	‐
Ipsilateral kidney mean dose (Gy)	12	12.4±6.1	11.9±6.8	0.25
Ipsilateral kidney $V_{15}(\%)$	20	28.2±18.6	31.5±20.0	0.06
Spinal cord D_max_ (Gy)	50	43.0±3.2	44.1±4.0	0.42
Liver mean dose (Gy)	24	14.8±6.4	14.6±7.9	0.19

a Significance (p<0.05)

$V_{x}(\%)=\text{volume receiving}\geq X\%\;\text{of prescribed dose}$; $D_{x}(\%)=\text{dose received by X}\%\;\text{of volume}$.

### D. Air cavities

**Figure 1:**
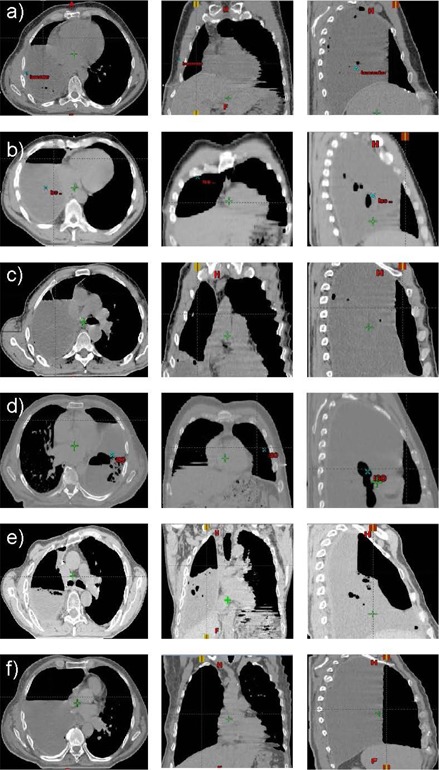
Air cavities remaining in the resected lung at the date of the planning CT in the axial (left image), coronal (middle image), and sagittal (right image) view. Five patients had a right‐sided MPM ((a), (b), (c), (e), (f)) and one patient had a left‐sided MPM (d). The volume of the air cavities are 150 cm^3^ (a), 225 cm^3^ (b), 243 cm^3^ (c), 276 cm^3^ (d), 379 cm^3^ (e), and 1276 cm^3^ (f).

Air cavity volume changes within the ipsilateral hemithorax following pleuropneumonectomy were measured as a function of time on various postoperative control computed tomographies (CT), RT planning‐CT. and CBCT.^(6)^ The air cavity volumes ranged from 150 cm^3^ to 1276 cm^3^. The axial, sagittal, and coronal views from the patients are displayed on Fig. 1. The evaluation of the variations of $D_{1},D_{99},V_{80},V_{85},V_{90},V_{95},V_{100}$, and $V_{105}$ with decrease of the air cavity volumes for the PTV1 and PTV2* were performed for IMRT and VMAT. Therefore, the dose distribution calculated on the planning CT was recalculated on all control CTs. When cone‐beam CTs or lateral images where available, the resected lung density of the planning study was modified in respect to these images. The effect of the air cavity decrease on the OARs was small as observed in this study and also on a previous study.^(6)^ Therefore, the OAR dose fluctuation and air cavity variation are not reported in this study.

### E. Statistics

Statistical analysis was performed using a paired *t*‐test. A p‐value of <0.05 was accepted as significant.

## III. RESULTS

The dose distribution calculated with AAA or Acuros, showed similar results. Indeed, $V_{90},V_{95}$, as well as the mean dose for the targets and air cavities, where within 1%. Therefore, all data presented were calculated with the algorithm, AAA, used clinically.

**Figure 2:**
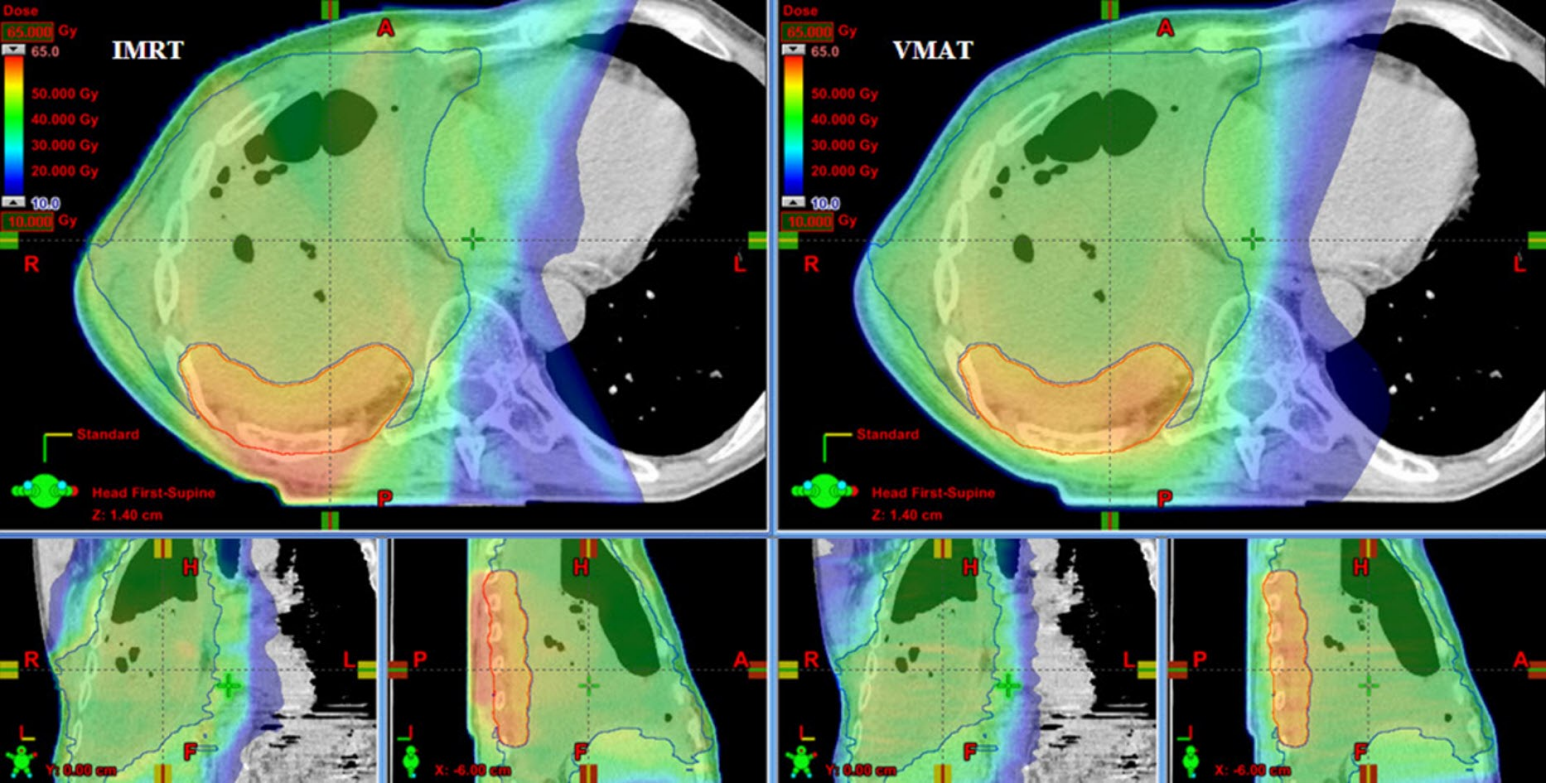
Typical dose distribution in axial sagittal and coronal planes for IMRT (left side) and VMAT (right side). Dose distribution is shown for the entire treatment to a total dose of 55.9 Gy to the PTV1 (red structure) and 45 Gy to the PTV2 (green structure) for the patient having the PTV1 volume closest to the mean PTV1 volume of all six MPM patients. The volume of the air cavities was 379 cm^3^.

A typical dose distribution for IMRT and VMAT are displayed in the axial, sagittal, and coronal view for one patient on Fig. 2. IMRT and VMAT covered PTV1 with the 95% of the prescribed dose, but the PTV2* was partially underdosed with IMRT. The dose to the spinal cord, contralateral lung, and heart is equivalent for this patient. A larger posterior dose from the PTV1 is observed for IMRT. This “hot spot” results from the intersection of the three dorsal fields.

### A. target volumes

DVH parameters for IMRT and VMAT plans for targets and OARs are summarized in Table 1. These parameters where calculated for VMAT using two partial arcs of 205°. The dose distribution was normalized to 55.9 Gy at 100% of the mean dose to the PTV1. The difference in dose homogeneity, minimal dose and maximal dose in the PTV1 for IMRT and VMAT was small and not significant. Indeed, the mean difference for $V_{95},V_{105},D_{1}$, and $D_{99}$ was within 1.8% (*p* not significant). Regarding the PTV2*, VMAT increased the $V_{95}$ by 0.5% and decreased $V_{105}$ by 1.8% (*p* not significant). $V_{80},V_{85}$, and $V_{90}$ for PTV2* were always larger than 98% for each IMRT and VMAT plans. Therefore, these values were not displayed on Table 1.

### B. OAR

Comparison data for OAR are summarized in Table 1. Both, IMRT, and VMAT were able to keep the dose to the OAR below the constraints fixed in Table 1, except for the ipsilateral kidney. The mean dose and $V_{15}$ for the ipsilateral kidney, which is surrounded by the target, could not be kept below 12 Gy and 20%.

The mean ipsilateral kidney dose and $V_{15}$ were 12.4 Gy and 28.2 Gy (respectively) for IMRT, and 11.9% and 31.5% (respectively) for VMAT (p=0.25 and 0.06). It was not possible to keep these values below our objectives of 12 Gy and 20% due to the target surrounding the ipsilateral kidney. For the contralateral kidney, both techniques were able to keep the mean dose far below the constraints of 12 Gy (3.6 Gy for IMRT and 3.8 Gy for VMAT, p=0.47), and $V_{15}$ was kept at 0% for IMRT and VMAT. The contralateral lung $V_{5}$ increased by 8.4% with VMAT in respect to IMRT, but the mean dose, as well as $V_{13}$ and $V_{20}$ to the contralateral lung, were reduced by 0.6 Gy, 2.7%, and 1.6% (respectively) with VMAT. The difference reached significance only for V13 and V20 (p=0.02). The maximal spinal cord dose as well as the mean liver dose for IMRT and VMAT were very close to each other and the difference was not significant (p=0.42 and 0.19).

### C. Mu and treatment time

The mean number of monitor units was drastically reduced from IMRT to VMAT by a factor 4.2 from 2080±414MU for IMRT to 485±82MU for VMAT in order to deliver 2.15Gy(p<0.01). The time required to deliver the plan was around 10 minutes for IMRT and 4 minutes for VMAT. The beam‐on time for VMAT depends on the number of monitor units, dose rate, gantry angle rotation, and gantry rotation speed. For all the plans performed with VMAT, the gantry rotation speed was always at its maximal speed, 4.8°/sec, and the dose rate was modulated accordingly. Therefore, in our case, the monitor units were not affecting the treatment time for the delivery of the VMAT plans, but only the gantry rotation angle determined the beam‐on time.

### D. Air cavities

**Figure 3:**
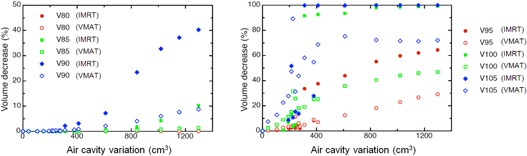
Impact of the air cavities volume variation on the PTV1 volume covered by ≥X% of the prescribed dose ($V_{x}$). Data are derived from six MPM patients with air cavities larger than 100 cm^3^.

**Figure 4:**
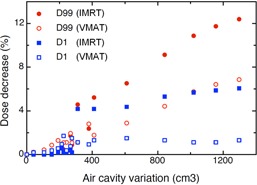
Impact of the air cavities volume variation on the minimal and maximal dose of PTV1 for IMRT and VMAT. The PTV1 minimal dose ($D_{99}$) and maximal dose ($D_{1}$) are defined as dose received by 99% and 1%, respectively, of the PTV1 volume. Data are derived from six MPM patients with air cavities larger than 100 cm^3^.

Air cavities on the planning CT are displayed in Fig. 1. The initial air cavity measured on the planning CT ranged from 150 cm^3^ to 1275 cm^3^. The air cavity remaining after EPP shrinks with time to disappear completely. The volume decrease can reach 220 cm^3^ in 12 days.^(6)^ The decrease of $V_{80},V_{85},V_{90},V_{95},V_{100},V_{105},D_{1}$, and $D_{99}$ in planning situation with air cavities are displayed on Figs. 3 and 4. IMRT and VMAT were not affected by the change of the air cavity volumes in respect to $V_{80}$. $V_{85}$ decreased only for IMRT when the air cavity variation was larger than 880 cm^3^. A decrease up to 10.2% for $V_{85}$ was observed when the air cavity volume decreased by 1276 cm^3^. IMRT (respectively VMAT) $V_{90}$ decreased when a variation larger than 310 cm^3^ (respectively 610 cm^3^) was observed. The decrease of $V_{90}$ was up to 40.3% for IMRT and 9.4% for VMAT. A reduction of $V_{95}$ for IMRT and VMAT was observed for air cavity variation $\geq 200\;{\text{cm}}^{3}$. The decrease of $V_{95}$ could reach 64.4% for IMRT and 29.2% for VMAT. A decrease ≥ 28% for $V_{95}$ is observed for IMRT in comparison to VMAT for air cavity reduction larger than 311 cm^3^. Concerning PTV2, similar results as for PTV1 have been observed when air cavity shrinks. $V_{80}$ (respectively $V_{85}$) was not affected by a change of air cavity for IMRT (respectively VMAT). Decrease larger than 20% of $V_{100}$ was observed when air cavity decreases by 300 cm^3^ and 600 cm^3^ for IMRT and VMAT, respectively.

A decrease of $V_{100}$ and $V_{105}$ was observed for IMRT and VMAT even for small volume shrinking (150 cm^3^). This decrease reached 100% for IMRT when the air cavity volume decreases by 310 cm^3^ for $V_{105}$ and 842 cm^3^ for $V_{100}$. For VMAT, a maximal decrease of 47% for $V_{100}$ and 72% for $V_{105}$ was observed.

The minimal dose and the maximal dose in the PTV1 as a function of the variation of the air cavities are displayed in Fig. 4. The minimal dose for IMRT decreased up to 12.4% and up to 6.9% for VMAT. The decrease of the maximal dose was more pronounced for IMRT with a decrease up to 6.1% and 1.7% for VMAT.

### E. optimal number of arcs for VMAt plans

DVH parameters and monitor units for plans performed with one, two, and three partial and full arcs are displayed in Table 2. These parameters have been calculated for the patient having the PTV volume closest to the mean PTV volume. When one arc is used, a full rotation showed better results than a partial rotation. Indeed, the $V_{95}$ for the PTV1 was increased from 93.3% to 95.6% and $V_{105}$ was decreased by 4.5% with the full‐arc rotation. All OARs had a lower dose with the full‐rotation arc, except for the maximal dose to the spinal cord. If two arcs are used instead of one arc, $V_{95}$ and $V_{105}$ for the PTV were slightly improved and the dose to all OARs was drastically reduced. The same observation was seen for the dose to the left and right kidneys, liver, heart, and maximal dose to the spinal cord. The difference between two arcs of 205° and two arcs of 360° was very small, with a small improvement for the partial arcs in respect to the lung dose. If the number of arcs is increased from two to three, there is a small benefit for $V_{95}$ for the PTV1. For the OAR, slight deviation was observed between two and three partial‐ or full‐arc rotation.

The number of monitor units increased with the gantry rotation angle. The monitor units ranged from 251 for a partial arc (205° rotation) to 708 for three full rotations (3 × 360°). The difference of beam‐on time between the partial‐arc rotation and full‐arc rotation was always 43% in favor of the partial‐arc rotation. Indeed, the gantry rotation speed was always at its $V_{x}(\%)=\text{volume receiving}\geq X\%\;\text{of prescribed dose}$; $D_{x}(\%)=\text{dose received by X}\%\;\text{of volume}$. maximal velocity, 4.8°/sec, during the rotation of the arcs for each plan. Therefore, the treatment time was not affected by the number of monitor units but was proportional only to the rotation angle performed by the gantry.

**Table 2:** DVH parameters and monitor units for plans performed with one, two, and three partial and full arcs. The values are calculated for one patient with the PTV1 volume closest to the mean PTV1 volume from all six MPM patients

	*1 Partial Arc*	*1 Full Arc*	*2 Partial Arcs*	*2 Full Arcs*	*3 Partial Arcs*	*3 Full* Arcs
$V_{95}(\text{PTV}1)(\%)$	93.3	95.6	94.5	95.1	95.1	95.6
$V_{105}(\text{PTV}1)(\%)$	6.6	2.1	2.2	1.8	2.0	1.9
$V_{95}(\text{PTV}2*)(\%)$	95.8	96.0	96.5	96.4	97.4	97.4
$D_{\text{mean}}$ ipsilat kidney (Gy)	10.8	10.6	6.4	6	6.6	6.2
$V_{15}$ ipsilat. kidney (Gy)	27.7	23.2	8.5	8.4	12	9.3
$D_{\text{mean}}$ contralat. kidney (Gy)	8.7	8.5	6	6.1	5.9	5.3
$V_{15}$ contralat. kidney (Gy)	8.6	5	0	0	0	0
$D_{\text{mean}}$ lung (Gy)	8.4	8.4	4.8	5.1	4.7	5
$V_{5}$ Lung (%)	87.1	87.1	30.3	36.1	29	33.3
$V_{13}$ Lung (%)	12.1	10.8	1.5	2.1	1.8	2
$V_{20}$ Lung (%)	0	0	0	0	0	0
$D_{\text{mean}}$ liver (Gy)	20.4	20.5	16.8	16.7	16.8	16.8
$D_{\text{mean}}$ heart (Gy)	17.7	16.4	11	11.4	10.8	12.2
$D_{\text{max}}$ myelon (Gy)	37.4	41.3	35.4	36.8	34	36.3
Monitor Units	251	298	513	638	563	708

a $V_{x}(\%)=$ volume receiving ≥X% of prescribed dose; $D_{x}(\%)=$ dose received by X% of volume.

## IV. DISCUSSION

Improved technologies enhance dose conformity, and avoiding dose delivery to critical structures has opened ways to treat complex oncological situations, such as MPM after EPP.^11^, ^12^ The RT treatment of MPM patients is commonly performed with IMRT, with improved dose conformity and homogeneity to the target in comparison with 3D conformal radiotherapy (3D CRT).^(5)^ However, a major drawback of IMRT is the treatment time. Treatment delivery is time‐consuming due to the large number of fields which are usually doubled due to split field technologies. Furthermore, a large number of MUs is needed (2080±414MU). The introduction of VMAT reduced the number of MUs by a factor of 4.2, reducing the treatment time from 10 minutes to 4 minutes. As reported previously,^(11)^ the decrease in treatment time reduces patient motion during the treatment delivery and thus results in greater agreement between the dose planned and dose delivered. This reduction in treatment time will decrease the time in which the patient has to stay in an uncomfortable position on the back with arms above the head.

Concerning the dose to the OAR, no major differences were seen between IMRT and VMAT, except for the lung. However, a reduction by a factor of 1.8 and 2.8 for $V_{13}$ and $V_{20}$, respectively, for the lung was observed for VMAT. This reduction of lung dose could decrease the risk of complication, such as radiation‐induced pneumonitis, where rates larger than 40% have been reported.^(12)^

The dose conformity and homogeneity were not statistically different for IMRT and VMAT. This is in agreement with previously published data.^(11)^ Nevertheless, the small difference between IMRT and VMAT concerning the target coverage and dose homogeneity on the planning CT does not imply an identical delivery of dose to the target during all treatment sessions. Indeed, air cavities remain in the chest after EPP. The air volume change during RT can be considerable.^(6)^ This will have a direct impact on DVH parameters for IMRT and VMAT plans. Indeed, when reduction $\geq \;311\;{\text{cm}}^{3}$ of the air cavity occurs, $V_{95}–V_{105},D_{1}$, and $D_{99}$ are drastically modified. The decrease of dose to the target can even reach 100% for IMRT for $V_{100}$ and 72% for VMAT. The reason comes from the fact that the air cavity volumes are always located on the ventral part of the resected lung. When the lung cavity is replaced by serofibrous tissue during treatment, dose distribution delivered with photons coming from the anterior direction will be most affected, and the proportion of photons coming from the anterior direction is more pronounced for IMRT than for VMAT. Therefore, a larger decrease of dose for IMRT in the target occurs when the air cavities disappear than for VMAT.

The overall decrease of dose coverage with decrease of air cavity is not a monotonic function, as observed on Fig. 3. These data are displayed for six MPM patients with air cavity larger than 100 cm^3^. The change of PTV1 dose coverage will be strongly influenced by the location of the PTV1. Indeed, the higher the proportion of photons depositing energy in the PTV1 going through air cavity before the PTV1, the higher the PTV1 will be affected by a change of air cavity.

A special concern comes with the decrease of air cavity volumes $\geq \;200\;{\text{cm}}^{3}$ reducing dose homogeneity of target volumes (Fig. 3(b)). Small air cavity decrease ($<\;200\;{\text{cm}}^{3}$) will impact only on the high dose in the PTV ($V_{105}$ and $V_{100}$). The minimal dose and $V_{95}$ will decrease by less than 2%.

If the air cavity volume decreases $>\;200\;{\text{cm}}^{3}$ compared to the planning situation, cold spots will appear in the target volume. Monitoring of air cavities can be achieved with two orthogonal kV images taken prior RT or with a CBCT. When an air cavity volume decrease larger than 200 cm^3^ is observed, a control planning CT might be helpful in order to assess the impact on the dose variation and the need for an eventual new treatment plan.

The dose distribution can be affected by the number of beams. Regarding VMAT, an improvement of the dose distribution was observed when two arcs are used instead of one arc. However, increasing the number of arcs from two to three did not lead to any further improvement for partial‐ or full‐arc rotation. When two arcs are chosen, partial‐arc techniques harbor the advantage to avoid dose delivery to the remaining lung, and the beam‐on time can further be reduced by more than 40% compared with the full‐arc rotation. Therefore, two partial arcs seem to be most suitable in respect to treatment time and dose distribution.

## V. CONCLUSIONS

VMAT using multiple partial arcs enhances the treatment quality with photons when compared to IMRT by reducing the dose of ionizing radiation to the remaining lung while saving treatment time and integral dose. VMAT dose distributions are less susceptible to changing air cavities than IMRT. It is recommended that patients having air cavity variation exceeding 200 cm^3^ be monitored attentively in order to consider adaptive replanning in case of structural changes during treatment.
